# Violence in the Nursing Workplace in the Context of Primary Health Care: A Qualitative Study

**DOI:** 10.3390/ijerph20176693

**Published:** 2023-08-31

**Authors:** Kisa Valladão Carvalho, Priscila Norié de Araujo, Felipe Lima dos Santos, Poliana Silva de Oliveira, Janaina Pereira da Silva, Karen da Silva Santos, Angelina Lettiere Viana, Cinira Magali Fortuna

**Affiliations:** 1 Public Health Nursing Graduate Program, Ribeirão Preto College of Nursing, University of São Paulo, Ribeirão Preto 14040-902, SP, Brazil; valladaokisa@gmail.com (K.V.C.); priscila.araujo@usp.br (P.N.d.A.); fel1pesantos@yahoo.com.br (F.L.d.S.); polianasilva@usp.br (P.S.d.O.); janaina.pereira.silva@usp.br (J.P.d.S.); karen.santos@usp.br (K.d.S.S.); 2 Laboratoire École-Mutations-Apprentissages, CY Cergy Paris Université, 92230 Gennevilliers, France; 3 Laboratoire Interdisciplinaire de Recherche sur les Transformations des Pratiques Educatives et des Pratiques Sociales, Université Paris-Est-Créteil-Val-de-Marne, 94010 Créteil, France; 4 Laboratoire Éducation et Diversité en Espaces Francophones, Université de Limoges, 87036 Limoges, France; 5 Public Health Nursing Graduate Program, Department of Maternal-Infant and Public Health Nursing, Ribeirão Preto College of Nursing, University of São Paulo, Ribeirão Preto 14040-902, SP, Brazil; angelina.lettiere@usp.br

**Keywords:** workplace violence, nursing professionals, primary health care, workplace health, nursing, violence

## Abstract

Violence demands considerable attention due to its complexity and social consequences. The objective of this study was to analyze violence in the nursing professional workplace in the context of primary health care in Brazil. It is a qualitative study with theoretical and methodological reference to institutional analysis. It was carried out in basic health units in Brazil. Nursing professionals (N = 11) participated in semi-structured interviews and discussion groups, in addition to a research diary and participant observation. Data collection took place from October to December 2021. The results are presented in five categories: types of violence and aggressors from the perspective of nursing professionals; the causes of violence reported by professionals; strategies for the management of violence; professionals’ proposals for preventing violence in health contexts; the consequences of violence in the workplace. Nursing professionals make up a large part of the workforce and have reported verbal, physical, moral, and psychological violence. The main causes are associated with user access to services. For the prevention of violence, professionals do not see themselves as protagonists of change. The consequences are the loss of quality of work and the health of professionals who requested sick leave and transfers. The study’s findings can help in the development of public policies and educational and management actions.

## 1. Introduction

Violence is not a recent phenomenon; it affects multiple areas of knowledge and demands considerable attention due to its complexity and social consequences [1]. It is a necessary field of study given the increasing demands in the public services, which significantly impacts people’s lives, mainly health workers [2].

Among the professionals most affected by the various forms of violence are nursing professionals [1], who make up a large part of the health workforce [3]. A study conducted in Macau, China, among doctors and nurses, shows that nursing was significantly at greater risk of physical violence as well as verbal aggressions and that patients were the main aggressors [4].

In a study conducted in Brazil with 1166 health professionals, 47.6% of the participants reported having been victims of violence during the COVID-19 pandemic. The study adds that, of the 130 (100.0%) nursing assistants who composed the sample, 92 (70.7%) had already suffered violence during their professional practice [5].

Despite the high levels of violence, most episodes are not recorded [6]. In a study conducted in Brazil during the COVID-19 pandemic, only 37.7% of health workers stated that their workplace had a procedure for reporting situations of violence and 68.4% stated that there was no incentive to report incidents [7]. A study conducted in Korea identified that of the 20% of interviewed health workers affected by physical violence, only 20.6% reported the fact to the service. Of the 61.2% of participants who suffered verbal violence, only 6.6% reported what had happened [8].

Violence perpetrated against health professionals can be classified in different ways. In the scientific literature, the criterion adopted is the nature of the violence, so it will be classified as psychological, verbal, sexual, or physical. Threats, for example, are a form of psychological violence characterized as verbal and/or gestural statements of intent to cause harm. Verbal harassment, in turn, is a form of verbal violence that is characterized as attacks against a worker’s sexual orientation, ethnicity, physical appearance, and/or skin color and can be manifested through swearing, shouting, and other disrespectful verbal interactions. Sexual harassment can include verbal and/or physical conduct of a sexual nature, such as insulting words, gestures, and touching. Physical assault is a form of physical violence, which can be manifested by the act of pushing, scratching, pinching, spitting, biting, hitting, pulling hair, throwing objects, slapping, kicking, stabbing, shooting, and any other physical action with the intent to cause harm [9,10].

The main causes of aggressions are delays in service provision, lack of human resources, and failure to meet patient and relatives’ expectations [11,12]. Violence results from an encounter between a perpetrator and a recipient. In the context of health services, the role of perpetrator can be occupied by different actors such as a patient, a patient’s family member, a non-family companion of the patient, a professional from the nursing team itself, a professional from another professional category, or a manager; even external factors such as policies and the work environment can occupy this role [6].

Violence perpetrated against health workers has been explored more in the context of emergency care units and hospital units [11,13]. However, in Brazil, as in several other countries around the world, a large part of the provision of care takes place within the scope of primary health care (PHC), but few studies approach the issue of violence in this context [12,14].

PHC is an organization of the health system’s proposition, adopted by many countries, which aims to offer comprehensive care to individuals and communities, aiming at continuous and longitudinal care with different actions comprising the population healthcare [14].

A comparative study of violence in both PHC and specialized services identified that verbal attacks were more present in PHC, while, in specialized care, more physical attacks occurred, with nursing teams being most exposed [15].

Experiencing violence at work has consequences on care and harms interpersonal relationships [6]. Such consequences are also seen in the health of workers and can lead to stress and poor performance [16,17,18].

In Brazil, the issue of violence against workers in PHC was examined in one study, but the phenomenon was analyzed from a quantitative analysis focused on verifying the correlation with the development of depressive symptoms [19].

There are no studies adopting a framework of Institutional Analysis (IA) to consider the violence to nursing professionals in PHC. The theoretical framework of IA aims to understand a social reality based on the practices and statements of its actors. The framework enables a better understanding of situations experienced in the daily life of an institution, including acts of violence [20].

Based on the lack of the literature on nursing workplace violence in PHC that adopts the theoretical framework of the IA approach to this issue, the research question of this study was: how do PHC nursing professionals in Brazil understand and deal with violence in the work environment? To answer this question, the aim of the study was to analyze violence in the nursing professional workplace in the context of PHC in Brazil.

## 2. Materials and Methods

### 2.1. Study Design

This was a qualitative study [21] that used the theoretical–methodological conceptions of Institutional Analysis (IA) [20]. The study was guided by the consolidated criteria for reporting qualitative research (COREQ) [22].

### 2.2. Theoretical–Methodological Framework

The theoretical–methodological framework of Institutional Analysis (IA) guided this study; it has previously been used in other studies in the primary health care setting [23]. The concepts: institution, analyzer, subject-group, and subject [20,24], in articulation with the data collection devices, allowed participants to reflect on their workplace context and the violence against health professionals, especially nursing professionals.

IA aims to provide processes of self-analysis and self-management, enabling the participating collectives to make changes, conduct new analyses, and take a leading role in the problematization of their demands and coping strategies, all important factors in the development of this study [20].

For IA, the institution is dialectically composed of three moments: instituted, instituting, and institutionalization. The institute has a static fixed characteristic and represents the norms, while instituting is dynamic and changeable. The institutionalization process is considered the third moment, which represents what we concretely see [20,24]. The institution, which differs from establishments and organizations, acquires dynamism through agents, who operationalize the institution through their daily practices [20,24].

The analyzer can be an event that points to, or even that expresses, the potential of change and puts in analysis the reality of the institution [25]. Resistance is understood as a social force that opposes another, called power, that is constituted in three moments: defensive, offensive, and integrative. The first refers to the conservative moment, the second corresponds to the revolutionary, and the last, composed with the institute, has an adaptive character. Resistance should be seen as a dynamic movement [25].

The subject group and the subjected group, on the other hand, refer to functioning positions of a certain group, not of a fixed character. The subject group refers to an operating mode of wider and autonomous communication that takes on the meaning of their practices, increasing their innovation and creation capacity. In contrast, the subjected group tends to adhere rigidly to their function and hierarchize relationships, blocking the ability to move and closing the opportunity for enrichment and change [26].

Therefore, the IA referential provides important support for the production of knowledge in the fields of nursing and general health because it values the entire research process, institutions, relationships, and health practices, considering the subjectivity of those involved [27].

### 2.3. Study Setting

The study was carried out in three primary health care units (PHCU) in Ribeirão Preto, an inland city of São Paulo State, Brazil, which has an estimated population of 720,116 inhabitants [28] and whose PHC network is composed of 47 units [29].

The city of Ribeirão Preto is administratively divided into five regions and each region has an agreement with a university. The health units included were selected according to the agreement established with the university of affiliation of the researchers and because they were classic PHCU where there was no family health strategy program or community health agent program.

### 2.4. Participants

Nurses, nursing technicians, and nursing assistants, the three professional categories that constitute the nursing workforce in Brazil [30], from PHCU participated in the study.

The choice to conduct the study with nursing professionals working in PHC was because they constitute a significant class of workers (both quantitatively and qualitatively) that make up the Brazilian unified health system; PHC is also the main gateway and organizer of longitudinal health care in Brazil [30]. In addition, nursing professionals (assistants, technicians, and nurses) have direct and continuous contact with the population served by the health services, a population that is often subject to vulnerable and stressful situations, factors that place nursing professionals in possible situations of violence in the work environment [30,31]. Table 1 shows the participant characterization.

### 2.5. Sampling, Sample Size, and Non-Participation

The PHCUs and participants were selected by convenience sampling in the city of Ribeirão Preto, São Paulo, Brazil. The study participants consisted of 11 nursing professionals from 3 different PHCUs. The selection of participants by convenience sampling was based on the researchers’ accessibility to the participants. This sample, however, may not be representative of the population at large, therefore this study can only be generalized to the population that was conveniently accessible, from which the sample was drawn [32].

The following inclusion criteria were adopted: being 18 years of age or older and having a cell phone or computer with internet access and a camera for virtual meetings.

An exclusion criterion was absence from work activities during the data collection period. It may be that some members of the nursing team were away from their work activities due to the experience of violence in the workplace. However, an investigation of the reasons for workers’ absence was not foreseen in the research protocol submitted to the research ethics committee and, therefore, was not investigated.

Refusals to participate in the study were justified by lack of time; the COVID-19 pandemic also interfered, as there were impediments to participate in this study due the high workloads of health professionals during the pandemic. The PCHU and professionals prioritized patient assistance to the detriment of participation in studies considering the pandemic context.

### 2.6. Data Collection

Data were produced through semi-structured interviews, participant observation, research diary, and discussion groups (Supplementary Materials, Link S1). The first author of this study carried out the data production, guided by the last author, as part of a research study at the master’s thesis level. The first and last authors, both female, have additional training in conducting interviews and groups and have participated in other qualitative research. The first author introduced herself to the participants as a psychologist and postgraduate student, emphasizing the purpose of the research.

Data were collected between October and December 2021 during the COVID-19 pandemic. Individual interviews were carried out, once with each participant, based on a thematic script addressing participants’ data such as age, gender, professional background, weekly workload, functions and activities performed in the health unit, aspects about the relationship with patients and team professionals, the understanding of violence in the work environment, the report of situations of violence experienced in the work environment, the impacts of violence on the worker’s relationship with other people and on the development of their work, and the report of strategies for prevention and/or management of situations of violence in the work environment. Nine interviews took place in the workplace in private rooms and two were conducted virtually through the Google Meet platform, with an average duration of 55 min. The interviews were audio-recorded and later fully transcribed.

Participant observation was carried out by the first author of the study, who was immersed in the work environment of the nursing professionals of the sites surveyed. This immersion helped to provide an understanding of the dynamics and institutional context of the health services surveyed, the presence of conflicts, the intensity of the relationships between the participants, the patients, and other classes of professionals that make up the health teams.

The dynamics of three PHCU were observed in different periods and days of the week, looking at the interactions between professional–professional, patient–professional, and patient–patient. Observations were registered in the research diary throughout the course of the research. For the IA, the research diary was a device that helped the researcher to record and describe the context, the perceived relationships, and the studied phenomenon [24].

The discussion groups were held after the individual interviews, which aimed to deepen the exploration of the theme of violence at work and, collectively, create proposals for the prevention of violence in PHCU through dialogue reflection. The activity lasted one hour and thirty minutes and five nursing professionals participated via the Google Meet platform.

Due to the social distancing requirements imposed by the COVID-19 pandemic, two participants opted to remotely conduct the individual interviews. The discussion groups also took place remotely through the Google Meet platform. In this present study, no significant differences were observed between the interviews conducted in person and virtually.

### 2.7. Data Analysis

The interviews and the discussion groups were audio-recorded and later transcribed in full. The data were analyzed through the contents related to the research objectives, using the notes in the research diary from moments of participant observation, being confronted together with some concepts of IA. From the data analysis process, five categories emerged. The data analysis was carried out by means of an exhaustive reading of the transcript of the interviews, groups, and field diary notes, seeking a deep contact with the material produced [33]. Units of meaning were identified and subsequently grouped into thematic categories, also relating them to the theoretical framework of IA.

In this study, the female gender was considered as a transversal aspect in the data analysis since this professional category in Brazil is made up mostly of women with significant impacts in the workplace and in the social context.

### 2.8. Ethical Aspects

The study was approved by the research ethics committee at the University of São Paulo at Ribeirão Preto College of Nursing under certificate of presentation for ethical consideration number 49693321.0.0000.5393 and report number 4.929.102, issued on 25 August 2021 in accordance with the guidelines and regulatory standards for research with human subjects, resolution number 466/2012 of the National Health Council of the Brazilian Ministry of Health [34].

An informed consent form was required for each participant. All participants were informed about the study objective, how their confidentiality would be protected, and their rights to withdraw from the study at any time. Those who agreed to participate gave written consent. The authors declared no conflicts of interest in this study.

To ensure the anonymity of the participants in the presentation of the results, they were identified by the following professional categories: “NUR” for nurses, “NT” for nursing technicians, and “NA” for nursing assistants, followed by the numbers from 1 to 5 and letters A, B, and C to categorize the primary health care units.

## 3. Results

The study participants consisted of 11 nursing professionals from 3 different PHCUs. The results showed that all participants suffered violence at some time in their professional practice in their workplace.

The analysis of the participants’ empirical data allowed the emergence of five analytic categories: (I) types of violence and aggressors from the perspective of the nursing professionals; (II) causes of violence indicated by the professionals; (III) strategies and management of the violence; (IV) proposed prevention strategies against the violence by the professionals in health contexts; (V) consequences of violence in nursing work.

### 3.1. Violence Types and Aggressors from the Perspective of Nursing Professionals

The interpersonal violence of psychological and verbal types was highlighted in patient reports. Ten professionals were affected by this type of violence; the main perpetrator, in the perception of the interviewees, was the patient:

[…] so she sent me to go fu** myself and told me that I am fat and lazy, understand? […].

(NT1, PHCU-A)

[…] called me a fag*, that hurts me so deep in my soul because I know that I am not a fag* […].

(NA1, PHCU-B)

Two cases of physical violence were also identified, directed against nursing technicians in a robbery situation by people carrying a firearm and a bladed weapon:

*[…] I was robbed inside the vaccine room, they put a knife in my belly and in the other [health provider] a gun […].*

(NT, PHCU-B)

The physical structure of the PHCU was also considered in the data analysis, e.g., the vaccine room where the mentioned robbery occurred was located in the unit entrance to optimize the access of people.

Another type of violence was the violence perpetrated by the team members themselves, cited by five participants, with the health units’ leaders and doctors as the main aggressors.

*[…] then she said “you are shameless”, she said this to me […] she tried to screw me on the work schedule at the time […].*

(NA2, PHCU-C)

*[…] when she [the physician] saw me, she started screaming in the middle of the hallway “you cheated” and so you see it’s not a joke […] It is creating problems with the schedule, delaying care for patients to complain about me, and she even called the manager saying […] that I was not doing my job.*

(NUR1, PHCU-C)

### 3.2. Causes of Violence According to Professionals

The participants considered that the motivator for violence by patients was the lack of medical appointments available.

*[…] not this week, the other one had it twice due to scheduling, once because there was no vacancy and the patient came out cursing […].*

(NA1, PHCU-B)

The lack of appointments without prior patient booking for acute conditions were also identified as a triggering factor for violence by patients.

### 3.3. Strategies and Management of Violence

In Brazil, all violence types, including violence that occurs against health professionals, should be obligatorily notified in the Brazilian health surveillance systems. In this present study, the compulsory notification of incidents of violence was not identified. However, professionals sought to record the violence perpetrated by patients to the team through annotations on their charts:

*[…] I made a record, and it is registered in the system […] if someone someday opens it, the nursing notes give the day, date, and hour.*

(NT1, PHCU-A)

Some professionals looked to the municipal civil guard as a means of protection against violent situations; nevertheless, in other units, it was seen that professionals were directed not to contact the formal security agencies, considering the guidelines of their supervisor:

*[…] we breathe and go, back to the drawing board, but we even have an orientation in general […] not to call the police, not to call the guard […] The recommendation was via the health unit manager […] “no, never mind”, “no, that is right”.*

(NUR3, PHCU-A)

Participants reported causes related to the lack of actions against events such as fear of the patient, in the belief that they would not lead to improvements, and also the presence of drug trafficking where the health unit was located:

*[…] she is the mother of people who from PCC (Primeiro Comando da Capital, the major criminal gang from Brazil and South America nowadays), understand? PCC members! So, it caused me discomfort […] so there is no one to help me in such a situation and no stance has been taken against her […].*

(NT1, PHCU-A)

### 3.4. Proposals for Preventing Violence against Professionals in Health Contexts

In the discussion group, based on the professionals’ knowledge and experiences, proposals were constructed and developed that, in the perception of the participants, would enable the prevention of violence in the units. The professionals came up with four main proposals that, according to them, would help prevent violence in the PHCU contexts. The first involved the “physical” security of professionals through surveillance cameras and security agents:

*[…] so, practical methods, cameras in the primary health care units […] outsourced security professionals or our municipal guards to inhibit that citizen […].*

(NT1, PHCU-A)

Regarding the second proposal, the participants reflected on the construction of group spaces for professional training.

The third and fourth proposals were the role of the government in raising awareness among the population on the role of health professionals and greater participation of the Regional Council of Nursing (COREN) in this space, encouraging and valuing the importance of nursing.

### 3.5. Consequences of Violence in the Nursing Workplace

Regarding the health of the professionals, in the interviews, reports of the consequences on the psychological aspects of the professionals and the sequelae of moral harassment were identified, including compromising the participants’ own perception of their professional competence as they sought to distance themselves from what happened, e.g., through medical sick leave.

*[…] so these are things that affected my pride, my professionalism, I started to feel very incompetent, I started to rethink […] it impacted my way of relating to her […].*

(NUR2, PHCU-B)

*[…] so in that aspect I felt a lot […] I think it is more than upsetting, I felt like garbage to be honest […] I even went to the doctor and he asked me if I needed sick leave […].*

(NA1, PHCU-B)

Thus, professionals have been transferred to other PHCUs and positions or even sought this as a way out of violence:

*[…] I came here, for example, transferred from another post, because the girl who was in the room […], she got into a physical fight with a patient […] the woman threatened to kill her and she was forced to get out of this unit.*

(NT1, PHCU-A)

Due to previous experiences of violence, some technicians do not take on certain care tasks and pass them on to the nurses:

*[…] when the person comes with this speech, I try to transfer the care to the nurse […].*

(NT3, PHCU-A)

*[…] I cannot resolve this, so I will transfer it to the nurse […] after this happened to me, I pass on all […].*

(NT4, PHCU-B)

There were modifications in the way they performed their tasks as functions were reconsidered based on previous experiences with violence situations:

*[…] I will administer the medication and a peripheral venous catheter, if I am emotionally shaken, I probably prefer to avoid doing this because it is a delicate procedure […].*

(NT1, PHCU-A)

## 4. Discussion

Verbal psychological violence stood out in the findings. For IA, this type of violence perpetrated by the patient can be seen as an analyzer that brings to light the conflicts and contradictions of the health units studied, since the existence of this form of violence allows analyzing the functioning of health services in PHC, revealing the lack of materials and supplies and shortages of professionals, aspects that make care precarious, leading to the problems observed in the units. Similar studies carried out in PHC point to a predominance of verbal psychological violence committed by patients against nursing professionals, which corroborates the present investigation [16,35].

Despite not being the most predominant type of violence in most studies, or which was not identified in this study, physical violence against the nursing team is present in everyday life and is also a result of the dissatisfactions of users listed above. Studies show the difference in percentage data in PHC and hospital sectors. Research carried out in Brazilian hospitals shows that 11% of nursing staff were affected by physical violence [36] and, in another study, physical violence affected 15.2% of health professionals [37].

In PHC, the results showed lower rates than those in the hospital sector, with the percentage of physical violence against nursing professionals being between 3.0% [38] and 8.5% [16]. A survey carried out with primary care health professionals in Madrid, Spain showed that 4.7% reported physical aggression and the main reason (with 36.1%) was dissatisfaction with the care received, which again relates to the general conditions offered by the primary sector [39].

One of the causes of violence is the predominance of medical appointments as the main action offered by health services. All work organization around the medical schedule [40] places the nursing staff in a position of the regulator of this scarce and insufficient resource to meet health needs.

For IA, the naturalization that the health of people is resolved with medical consultations, prescriptions of medications, and measures of good living, produces simplified explanations for the phenomenon of violence, blaming and individually identifying the aggressors (in this case, patients and colleagues).

In this regard, violence is an analyzer of the contradiction in the organization of PHC services that should operate with an expanded logic of the health and disease process, considering the social determinants and conditions and not just the disease and its medicalization.

The literature highlights that the lack of available medical appointments is related to the high rate of patients’ missed appointments, which reaches 19.2% of recorded missed appointments, which intensifies the schedules and increases the waiting time for new consultations [41,42]. On the other hand, Brazilian public services have been facing cutbacks in public funding, especially in the health area, reflecting a reduction of material resources, equipment, and hiring of personnel. In general, public health services are operating, for the most part, without the necessary resources to meet the population demand, directly impacting the professional practices, generating dissatisfaction with the service quality, and producing the phenomenon of violence [43,44].

Problems related to health care, such as problems in the organization of services, poorly protected environments, and a shortage of professionals, centered on a curative unresolvable care model, hinder the process of welcoming patients and establishing a therapeutic and harmonious relationship between health professionals and patients. Nursing professionals, because they have direct and continuous contact with patients and their companions, are more susceptible to situations of user discontent and suffer inappropriate treatment such as verbal aggression, threats, and blame for delays or precarious conditions of care [45].

It is possible to infer that verbal violence also goes through a process of naturalization, which prevents the phenomenon from being confronted [46,47]. This may be related to the fact that this form of violence has been seen as more socially accepted as it causes less visible damage and lack of physical harm [47] and also because it is considered to be expected in the context of public services.

Otherwise, unit managers play important roles in mitigating the problem, although, in this study, it was possible to observe reports of moral harassment committed by themselves against nurses and technicians. As reported by NUR2-PHCU-B, it is noted that the manager understands the role of the nurse as “leading” and “commanding”, established in the nursing practice, unlike what is attributed to the professional in the management of teams. The different logics of seeing the management, on the part of the other health professionals, are causes of conflicts, as different actions are expected in the management of the nurses [46,48].

As measures to protect themselves, nursing professionals try to remain silent or ask for help from co-workers. This deserves reflection, since, by using individual ways to protect themselves, situations can be trivialized and crystalized in health institutions as commonplace. Thus, it is necessary to collectively seek alternatives, involving workers and managers, among others [49].

The violence against nursing staff expresses the cultural, educational, and social context of an unequal country with a history strongly marked by the colonial and patriarchal process [50]. In this sense, verbal violence with offenses related to sexual orientation and physical appearance (obesity) are expressions machismo and an imposition of standards of ideal bodies present in Brazilian society. Physical violence against nursing teams is related to the location of the rooms. The vaccine room (where the theft took place) is located near the entrance to the unit, which is a room that provides access and circulation of people close to the entrances to the services, following the guidelines and standards of the physical premises of the PHCU, aimed at access for patients. However, the lack of security favors the exposure of these professionals. A study identified that nursing professionals feel less secure than other health professionals due to their direct relation with the public [51]. Their perception of the environmental conditions directly impacts their care when they feel their security is threatened [51].

Another type of violence reported was moral harassment, with the main perpetrators mentioned being doctors and service leaders. Studies corroborate these data; the medical team has been the professional category most cited for moral harassment against the nursing team, responsible for 81% and 92.62% of reports of moral harassment [47,52].

In this study, the relations of power and knowledge between medicine and nursing were strongly marked. The medical team is the professional category most cited for this type of verbal psychological violence, which is configured as moral harassment and is suffered by nursing staff in some studies [53,54]. One study [38], for example, highlighted that physicians were responsible for 84.2% of reports of moral harassment in a PHC location in a Brazilian municipality. Another study carried out in Brazil showed even higher levels, characterizing 92.7% of medical professionals [55].

In terms of IA, the historical differences between nursing and medicine may be behind these data, as both institutions, as seen by the framework, are marked by territorial disputes. Nursing has been strictly placed under the authority and orders of priests and doctors [23]. Similarly, a study shows that, from the perspective of other professionals, nursing is still hierarchically submissive to other professions, with its aspects linked to care and the feminine and linked to the conception of domination between men (medicine) and women (nursing) [56]. Another study also conducted in Brazil found that the power relations between nurses and doctors have other associations, including salary differences. Therefore, there is supposedly a social and hierarchical prejudice that afflicts nursing, since doctors are seen as having more authority than nurses for presenting themselves as proponents of cures, medicalization, and having higher salaries [47,57].

In relation to strategies for managing violence, the results of this study showed that all professionals suffered some type of violence, but there was no formal notification action. Of the professionals who sought to record violence by the patient, the resources used were annotations in medical records.

The scientific literature indicates the underreporting of records of verbal psychological violence, with rates reaching 5.6% of cases [58]. Another investigation did not identify measures taken against the aggressors and the reactions were to report the violence only to colleagues and managers [16]. From this perspective, underreporting data can be called a resistance movement according to IA [25]. In this case, as an integrative resistance, which protects the instituted in order to preserve it, that is, a notification is not made to stop the violence because this would reveal organizational and relational contradictions that could generate changes in the organization.

The performance of those responsible for the services is fundamental in confronting violence by supporting the teams with appropriate measures, which was not identified in the interviewed statements. On the other hand, this study identified that organized crime can be an obstacle in the search for resources in general; the presence of the police or municipal guard generates insecurity in units; so, for fear of conflicts with family members of drug traffickers, they are no longer a used measure, as found in another study [59]. Another way of dealing with the situation of violence against the aggressor patient is to resort to other professionals for joint care, which the literature points to as a measure that impacts the reduction of violence and expresses the importance of being able to count on the team in moments of conflict [60].

In the discussion group, in view of the analysis of the participant proposals, it was identified that they were similar to those indicated in the literature, such as safety measures in the workplace, including cameras and security agents, the necessity of professional training [61], governmental participation [6], and the nursing council. Otherwise, from the IA perspective, a verticalization and hierarchization of the decisions were observed, that is, the group crystalized its structure and its determinations were directed externally in the search for resources and other professionals to face the violence, showing little autonomy from reality itself, indicating what the referential calls a subjected group [26].

The consequences of violence and moral harassment identified in this study are similar to those found in another study that describes the consequences of this phenomenon seen in professionals affected with feelings of incompetence regarding their work, as well as demotivation and fear [52]. Numerous workers who experience moral harassment or other violence in the workplace look for ways to avoid the real problem in the absence of institutional actions, such as removals and work transfers (the latter being seen as the common resolution of problems), but this turnover interferes negatively in the dynamics of the services, requiring new professionals to learn the role and breaking the bond with the population [17].

During the COVID-19 pandemic, nurses faced high levels of violence and harassment due to the environment of stress and uncertainty, which exacerbated the already existing tensions in the health workplace. Whatever its forms, violence in the workplace can negatively affect the physical and mental health of nursing professionals in addition to compromising the quality of patient care [38,62].

Violence against nursing staff during the COVID-19 pandemic has been widely recognized as a serious problem; many countries are still working to address this issue. It is critical that healthcare institutions implement safety and security measures for nursing personnel and provide the necessary training and resources to help them cope with pandemic-related violence and stress [63].

Thus, this study identified that violence also modifies the offer of assistance in the service so that, as a result of previous negative experiences, professionals avoid certain procedures and refer them to the nurse, for whom taking on the care of other professionals reduces the management time of the nursing team and patient care [64]. The nurse has an essential role in health contexts; in addition to those mentioned, a study showed that the excessive workload of nurses leads to mental and physical illness, being the main reasons for accidents at work, exhaustion, and medication errors. In addition, their inefficiently expanded occupations result in negative effects on quality care. This professional is also responsible for the coordination and administration of health services [61].

## 5. Conclusions

The results showed the presence of violence in nursing work and that verbal violence was the most mentioned in this study, attributed to patients, followed by the other health providers, including physicians and service managers, and physical violence was committed by people in the community. The main causes of violence involved lack of medical appointments and unscheduled consultations for acute cases.

The strategies found in the face of violence were to record violent incidents in the patient medical records. The management modes proved to be an important point for dealing with violence. Organized crime was one of the obstacles that prevented professionals from reporting violence. The prevention proposals noted the difficulty of professionals to be protagonists of their own reality as they sought the solution in external agents. Violence had a negative impact on mental health, as, in order to prevent the violence, the response involved removal, transfers, or changes in procedures and functions, increasing the workload of the nurses.

It is known that, historically, nursing is a predominantly female profession, with a social association related to violence in the workplace. Thus, by conducting research on this professional category, traits of social behavior with women in the social context in which they are inserted were also investigated.

One of the limitations involved the non-participation of other professional categories and also the non-participation of patients as perpetrators of violence identified by nursing professionals participating in this research, thus raising new research from this perspective of participants and data analysis. The COVID-19 pandemic also had an important impact in this study regarding the sampling of participants and the data collecting. It is suggested for future research that a higher number of nurses be included and an analysis that considers the markers of ethnicity and gender (since they were not covered in this study).

Also, a suggestion for future research is to explore the measures for confronting violence against health professionals by holding discussion groups and reflections with more than one meeting. For health services, spaces for dialogue and reflecting on the violence that permeates the daily lives of health professionals are suggested, considering their interpersonal and interprofessional relationships (as there is a need to expand these moments of reflection) and even co-management for the consolidation of coping strategies.

## Figures and Tables

**Table 1:** Characterization of participants of the study.

Code	Age	Gender	Professional Category	Years Employed	Years Employed in the PHCU	Dual Employment
NUR3-PHCU-A	30	F	Nurse	9 years	1 year 5 months	Yes
NT2-PHCU-A	39	F	Nursing technician	17 years	1 year 10 months	No
NT3-PHCU-A	28	F	Nursing technician	6 years	2 months	No
NT1-PHCU-A	43	M	Nursing technician	23 years	4 years	No
NUR2-PHCU-B	43	F	Nurse	18 years	1 year 10 months	No
NT5-PHCU-B	44	F	Nursing technician	24 years	4 years	Yes
NT4- PHCU-B	41	M	Nursing technician	17 years	5 years	Yes
NA1-PHCU-B	57	F	Nurse assistant	35 years	10 years	No
NUR1-PHCU-C	34	F	Nurse	6 years	6 years	No
NA2-PHCU-C	54	F	Nursing assistant	17 years	7 years	No
NA3-PHCU-C	61	F	Nursing assistant	16 years	8 years	No

NUR—nurse; NT—nursing technician; NA—nursing assistant; PHCU—primary health care unit; F—female; M—male.

## Data Availability

The data presented in this study are available on request from the corresponding author. The data are not publicly available due to confidentiality and privacy concerns.

## Supplementary Materials

The following supporting information can be downloaded at: https://www.mdpi.com/article/10.3390/ijerph20176693/s1, Link S1: semi-structured interviews script, participant observation script, and discussion groups script.
