# Supplementary material for: Violence in the Nursing Workplace in the Context of Primary Health Care: A Qualitative Study
Source: Int J Environ Res Public Health. 2023 Aug 31;20(17):6693. doi: 10.3390/ijerph20176693 (PMC10487648; doi:10.3390/ijerph20176693)
Supplement: Supplementary file 1 [file ijerph-20-06693-s001.zip › ijerph-2449805-supplementary.pdf]

NUPESCO
Núcleo de Pesquisas e Estudos em Saúde Coletiva
Profa. Dra. Maria Cecília Puntel de Almeida

FAPESP
FUNDAÇÃO DE AMPARO À PESQUISA DO ESTADO DE SÃO PAULO

Date:____/_____/______ ID code:____________

DATA COLLECTION SCRIPTS

PARTICIPANT OBSERVATION SCRIPT OF BASIC HEALTH UNITS FOR RECORDING IN THE RESEARCH DIARY

Unit:

Date:

Time:

Observe the routine of the unit regarding:

Start of activities and development of activities in the unit.

Types of care, change of shifts of professionals, most frequent dialogues, issues that cause discomfort between workers, workers and users, meetings, way of solving problems.

SEMI-STRUCTURED INTERVIEWS SCRIPT - HEALTH PROFESSIONALS

Date of interview: __________ Interview number: ______

Name:

Sociodemographic data of the health professional:

Age: ( ) male ( ) female

Gender: ( ) male ( ) female

Marital status:____________

Do you have children? ( ) no ( ) yes. If yes, how many:____________

Professional training:_____________

Weekly workload:__________________

Do you have another employment relationship? If yes, which one?

Postgraduate degree: Yes ( ) No ( ). If yes, which:________

Length of time working in health:_________________

Functions performed in the unit: _________________

Tasks you perform most often: ______________________

Age range of participants you have most contact with:________________

Are you affiliated to the health professionals' union? ( ) no ( ) yes

Topics:

Personal life:

- I would like to know a little about you; tell me about the place where you were born and grew up; about your family of origin and its most striking characteristics.
- Tell me about your experience with sports, religious, and cultural practices.

Professional life:

1. Tell me a little about your daily life in the unit.
2. How do you consider your relationships with the users of this unit?
3. And how do you consider your relations with the professionals of the team? Tell me a little.
4. Have you ever been affected by any professional illness? Have you ever needed to leave for any reason? Have you ever had to change units? If so, why?

In view of our research objective, in which we want to study the meanings of violence constructed by nursing professionals, I would like to know:

5. What is violence for you?
6. What do you consider to be violence in the health context?
7. Have you ever experienced any situation at work?
8. If you have already experienced violence in the unit, could you tell me how it happened?

Did these situations you experienced make you modify/change the way you relate to a professional/user, or even the way you perform a procedure to avoid these situations?

Do you think that violence impacts your way of working? If so, how?

9. What causes do you attribute to violence in health?
10. Do you know health professionals who have suffered, practiced, or practiced some act that you consider violence? Tell me more about it.
11. In your opinion, what could be done to prevent violence in the unit?
12. And how could you prevent violence in the unit? What proposals would you have?
13. Is there anything else you would like to talk about that we have not already discussed?

DISCUSSION GROUPS SCRIPT

Group contract: ask for consent for the recording of the meeting from the group participants, emphasizing that the speeches will be used for research, respecting the anonymity of all present; request the punctuality of the meeting, as well as respect for the speech of the other and confidentiality of the information produced in the conversation rounds.

Orientations: remind participants that the time allotted for the activity is approximately one-and-a-half hours (90 minutes).

Ask the group to introduce themselves.

Introduce the objective of the round table: in this meeting, we are here to discuss, reflect a little more on the problem of violence.

1. What do you consider to be violence at work?

2. Do you think it is possible to prevent violence within the Basic Health Unit where you work? If so, what proposals would you have?
